# Case Report: Neurological and pulmonary angiostrongylosis in a Ryukyu long-furred rat (*Diplothrix legata*) on Amami-Oshima Island, Japan

**DOI:** 10.3389/fpara.2026.1797945

**Published:** 2026-04-10

**Authors:** So Shinya, Toshihiro Tokiwa, Keita Sakashita, Hisashi Yoshimura, Ryotaro Suzuki, Ryouta Torimoto, Yukinori Muraoka, Masami Yamamoto

**Affiliations:** 1 Amami Wildlife Medical Center, Amami, Kagoshima, Japan; 2 Laboratory of Veterinary Parasitology, School of Veterinary Medicine, Nippon Veterinary and Life Science University, Musashino, Tokyo, Japan; 3 Laboratory of Physiological Pathology, School of Veterinary Nursing and Technology, Nippon Veterinary and Life Science University, Musashino, Tokyo, Japan; 4 Yuinoshima Animal Clinic, Amami, Kagoshima, Japan; 5 Veterinarian Medical Development, Adachi, Tokyo, Japan

**Keywords:** endangered animals, pathology, rat lungworm, Ryukyu long-furred rat, wildlife, zoonosis

## Abstract

A Ryukyu long-furred rat (*Diplothrix legata*) rescued on Amami-Oshima Island, located in the southwestern Japan, exhibited neurological signs, including nystagmus and ataxia, and died six days after supportive treatment. Gross and histopathological examinations revealed nematodes in the brain, lungs, and heart. In the meninges, immature worm sections were associated with marked inflammatory thickening. Pulmonary lesions included adult worms, eggs, and larvae accompanied by hemorrhage, edema, thrombosis, and extensive necrosis with concurrent *Aspergillus* infection. Mitochondrial cytochrome *c* oxidase 1 sequences obtained from the nematodes identified the ac1 lineage of *Angiostrongylus cantonensis*, a haplotype previously reported from mainland Japan and Amami-Oshima Island, but distinct from the lineage common on the neighboring islands of Okinawajima and Tokunoshima. This case supports the occurrence of angiostrongylosis in the Ryukyu long-furred rat and suggests that this species may manifest both neurological and respiratory disease manifestations. Further epidemiological investigations targeting invasive rodents of the genus *Rattus*, intermediate host, namely terrestrial gastropods (e.g., snails and slugs), and potential paratenic hosts (e.g., amphibians and reptiles), are warranted to clarify the transmission dynamics of *A. cantonensis* within the Amami Islands and to assess its potential impact on this endangered endemic rodent.

## 1 Introduction

The Ryukyu long-furred rat, *Diplothrix legata* (Rodentia, Muridae), is an endemic murine species restricted to three islands in the Ryukyu Islands in southwestern Japan (Iwasa, 2015). Owing to its limited distribution and ongoing population decline, it is currently listed as Endangered (EN) by the IUCN (Watari, 2025). Previous studies have reported *Angiostrongylus cantonensis* (Nematoda, Metastrongylidae) infection in individuals from islands of Okinawajima (Nakaya et al., 2013; Okano et al., 2014) and Amami-Oshima (Tokiwa et al., 2020) with associated pulmonary lesions, suggesting that this species is susceptible to pulmonary angiostrongylosis (angiostrongyliasis). Although *A. cantonensis* has been detected in the brain of a Ryukyu long-furred rat from Tokunoshima Island, the associated inflammatory changes were mild, and the observed neuropathology was considered more likely attributable to a cerebral lymphoma rather than to the parasite itself (Sakashita et al., 2025). Nevertheless, the parasite is known to migrate to the central nervous system in other rodents (Griffin et al., 2025), where it can cause severe neurological disease. Understanding the ecology and host range of this parasite is therefore important for evaluating potential threats to insular rodent populations.

Here, we report a case of a Ryukyu long-furred rat exhibiting neurological symptoms, in which *A. cantonensis* infection was detected directly within the meninges. This represents the first confirmed case of neuroangiostrongylosis in this endangered species. This observation highlights the potential for severe clinical outcomes and emphasizes the importance of monitoring parasitic threats for the conservation management of insular mammals. Our findings contribute to a better understanding of host–parasite dynamics of *A. cantonensis* in the Ryukyu Islands and underscore the need for continued surveillance to assess its impact on vulnerable endemic species.

## 2 Case description

**Figure 1:**
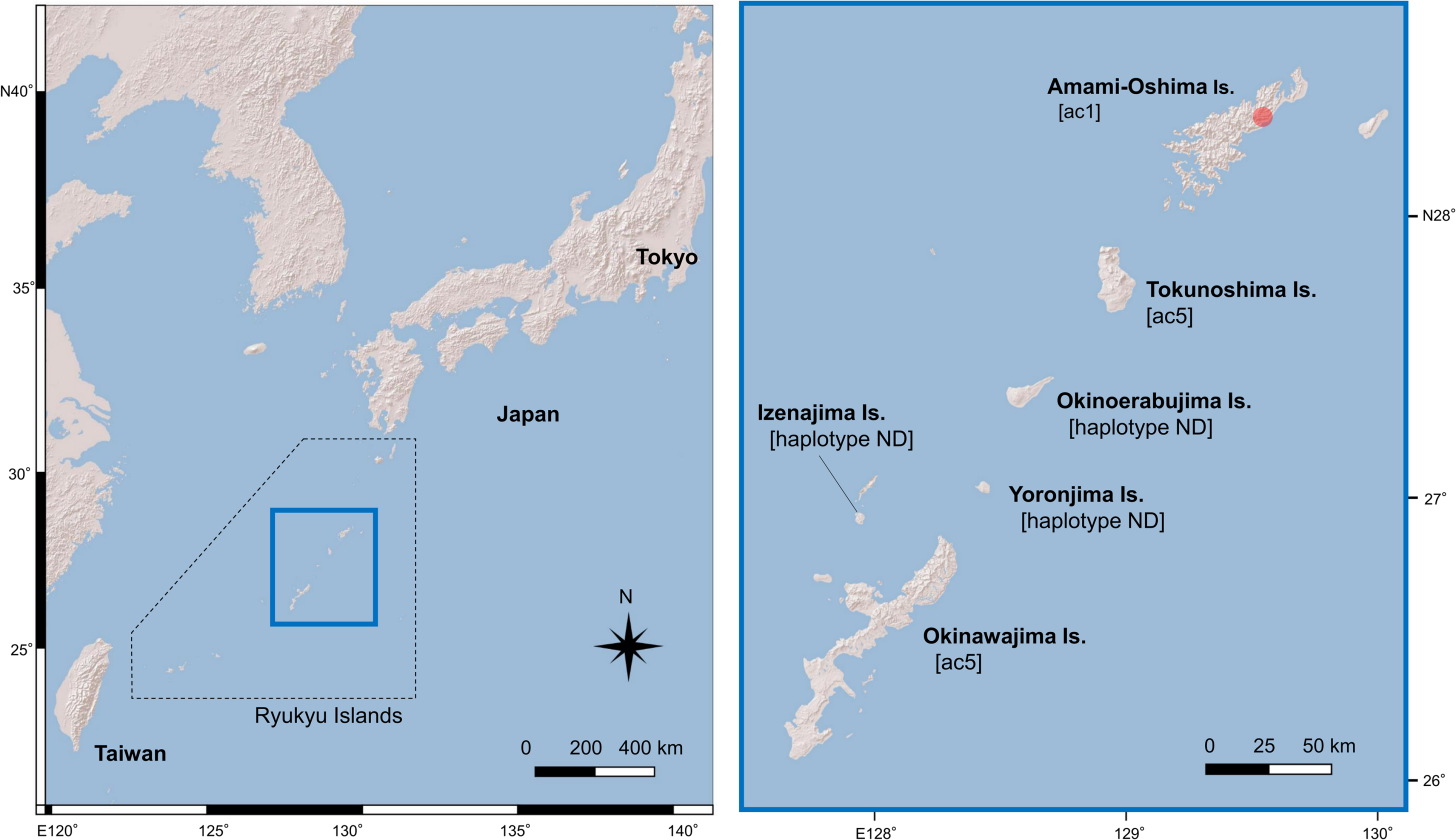
Map of the Ryukyu Islands showing the three islands where the Ryukyu long-furred rat (*Diplothrix legata*) is endemic: Amami-Oshima, Tokunoshima, and Okinawajima Islands. The red circle indicates the location where the case was identified. Brackets indicate previously reported *Angiostrongylus cantonensis COI* haplotypes, and ND denotes not determined.

On July 17, 2024, an adult female Ryukyu long-furred rat weighing 352 g and exhibiting ataxia was found in front of a private residence in Naze Oaza Itsubugachi, Amami-Oshima Island (**Figure 1**), and was rescued and subsequently transported to the Amami Wildlife Medical Center.

Clinical signs included horizontal nystagmus, ataxic gait, labored breathing, polydipsia, polyuria, and myiasis within the vaginal cavity. No external injuries were observed, and radiographic examination revealed no fractures. Blood test results were within normal limits, including the total white blood cell count, and no abnormalities were detected in the peripheral lymph nodes. Supportive treatment consisted of oxygen supplementation; enrofloxacin 10 mg/kg subcutaneously every 12 h; prednisolone 1 mg/kg subcutaneously every 24 h; metoclopramide 0.5 mg/kg subcutaneously every 12 h; and subcutaneous fluid therapy with lactated Ringer’s solution 20 mL/day. Despite these interventions, the animal became progressively weaker, and its neurological condition deteriorated, resulting in death on July 23, 2024.

## 3 Diagnostics assessment

### 3.1 Morphological observation

Necropsy was performed at the Amami Wildlife Medical Center, and major organs (brain, eyes, lungs, heart, liver, spleen, kidneys, and intestines) were fixed in 10% formalin. The tissues were routinely processed for histopathological examination at Nippon Veterinary and Life Science University.

**Figure 2:**
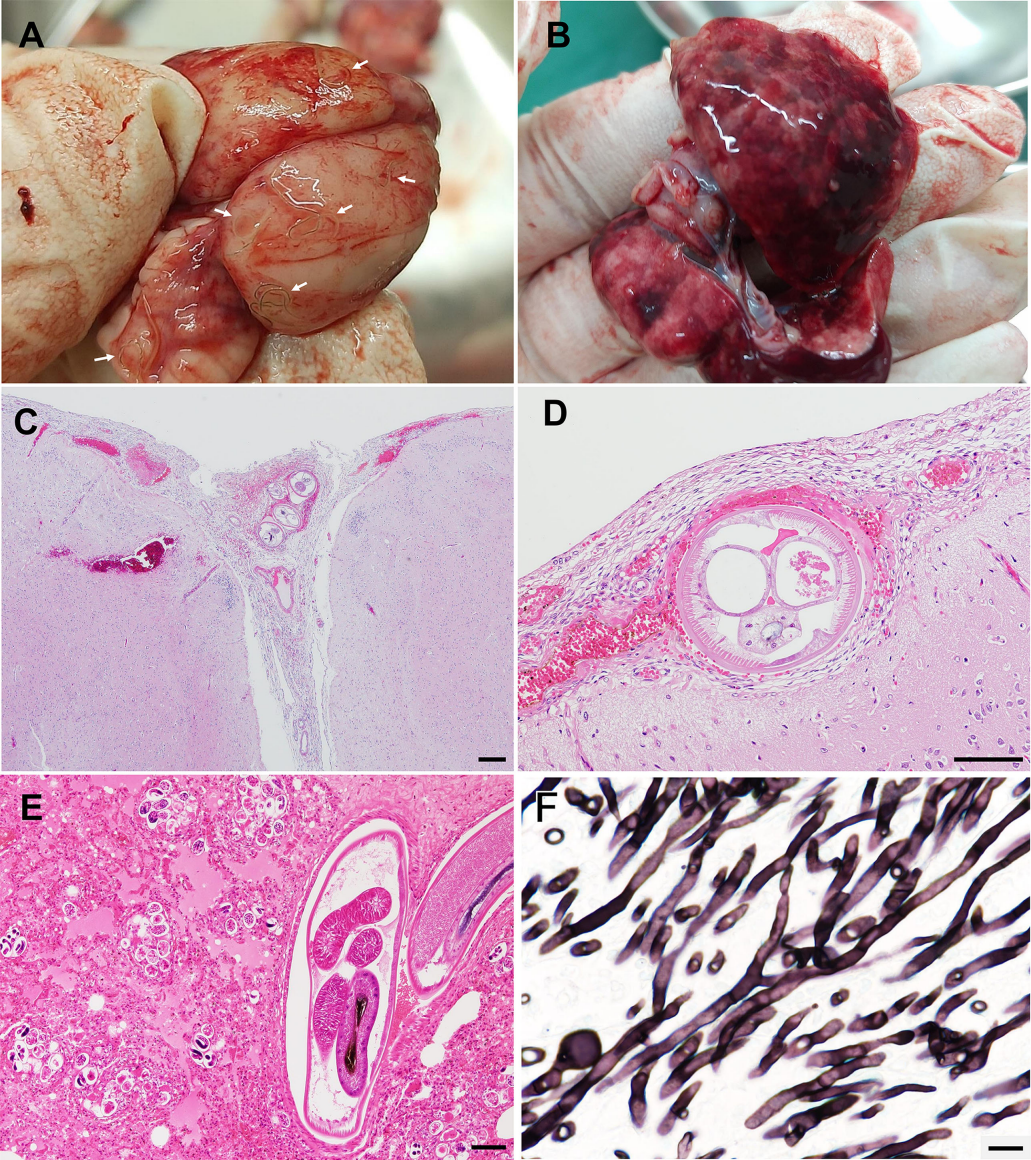
Gross **(A, B)** and histopathological **(C–F)** findings in a Ryukyu long-furred rat (*Diplothrix legata*) from Amami-Oshima Island, Japan. **(A)** Brain showing hemorrhage and nematodes (arrows). **(B)** Lungs showing red mottling. **(C)** Brain. Cross-sections of nematodes accompanied by hemorrhage and thrombi are observed in the meninges. Hematoxylin and eosin (HE). Scale bar = 200 μm. **(D)** Meninges containing cross-section of nematode. HE. Scale bar = 100 μm. **(E)** Lung containing eggs, larvae, and adult nematodes. HE. Scale bar = 100 μm. **(F)** Fungal hyphae in the lung. Grocott’s methenamine silver stain. Scale bar = 10 μm.

Gross examination revealed hemorrhage on the surface of the brain (**Figure 2A**), with a total of seven nematodes present on the cerebral surface and within the meninges. The lungs (**Figure 2B**) were reddish and exhibited nodular lesions, from the cut surface of which numerous nematodes were observed. In the heart, two nematodes were found within the right ventricle. Histologically, the meninges (**Figure 2C**) exhibited hemorrhage, congestion, edema, and fibrous thickening, and thrombosis, with a small number of infiltrating macrophages but without evident eosinophilic infiltration. Cross-sections of nematodes (**Figures 2C, D**) measuring 180–250 µm in diameter were present within the leptomeningeal tissues. The nematodes (**Figure 2D**) exhibited distinct lateral chords, coelomyarian–polymyarian musculature, a multinucleated intestinal epithelium, and immature reproductive organs, resembling those previously reported for *A. cantonensis* (Sakashita et al., 2025). The lungs exhibited diffuse accumulation of blood and serous fluid within the alveolar spaces (alveolar edema and hemorrhage). Numerous cross-sections of nematodes, including adults, larvated eggs, and larvae (**Figure 2E**) were present within the lumina of the pulmonary arteries and adjacent parenchyma. Adult worms retained the same fundamental morphological features as the immature nematodes described above but had fully developed reproductive systems. Female adults measured approximately 220–300 µm in cross-sectional diameter and possessed two mature reproductive tracts, whereas males measured 190–240 µm and possessed a single male reproductive tract. Scattered thrombi were present within the pulmonary arteries, many of which were accompanied by adjacent nematode cross-sections. Extensive regions of the pulmonary parenchyma exhibited coagulative necrosis accompanied by infiltration of moderate numbers of neutrophils. Within these necrotic foci, filamentous fungi were markedly proliferative. Filamentous fungal elements were also commonly observed within thrombi in the pulmonary arteries. Grocott’s methenamine silver staining revealed slender, septate, acutely branching hyphae (**Figure 2F**) consistent with *Aspergillus* spp. (Ascomycota, Eurotiales). In the heart, several cross-sections of adult nematodes were observed within the lumina of the pulmonary arteries.

### 3.2 Molecular analysis

DNA was extracted from lung tissue, and three worms obtained from the brain, pulmonary artery, and heart using the QIAamp DNA Mini Kit (Qiagen, Germany), following the manufacturer’s instructions. PCR amplification was performed to detect fungal DNA and identify the nematodes. For fungal detection, the nuclear ribosomal RNA region, including the full lengths of internal transcribed spacer 1 (ITS1), the 5.8S rRNA gene, and ITS2, was amplified using the primer pair ITS1 (5′-TCCGTAGGTGAACCTGCGG-3′) and ITS4 (5′-TCCTCCGCTTATTGATATGC-3′) (White et al., 1990). For nematode identification, the mitochondrial cytochrome *c* oxidase 1 (*COI*) gene was amplified using cox1F (5′-TTTGTTTTGATTTTTTGGTC-3′) and cox1R (5′-AGGATAAATCTAAATACTTACGAGGA-3′) (Tokiwa et al., 2012). The PCR reaction mixture consisted of 20 µl volume containing 0.2 μL of Ex Taq DNA polymerase (Takara, Japan), 2.0 μL of 10× Ex Taq buffer, 1.6 μL of dNTP mixture, 0.2 μL of each primer (50 µM), 1.0 μL of DNA template, and distilled water to a sinal volume of 20 µL. Distilled water was included as a negative control in all reactions. PCR amplification of the fungal gene was performed with an initial denaturation at 95 °C for 5 min, followed by 35 cycles of 95 °C for 1 min, 50 °C for 1 min, and 72 °C for 2 min. Amplification of the *COI* gene was carried out with an initial denaturation at 94 °C for 2 min, followed by 35 cycles of 94 °C for 30 s, 60 °C for 30 s, and 72 °C for 2 min. PCR products were directly sequenced by Eurofins Genomics (Tokyo, Japan) or Macrogen Japan (Tokyo, Japan). The obtained sequences were aligned using MAFFT ver. 7.490 (Katoh and Standley, 2013) implemented in Geneious Prime (ver. 2025.0.2, Biomatters Ltd., New Zealand), and sequence similarity was assessed using the NCBI BLAST program.

*COI* sequence analysis revealed that three worms collected from the brain, lungs, and heart were genetically identical. A representative sequence (589 bp) showed complete identity with the *A. cantonensis* ac1 haplotype (Accession no. AB684358). This lineage has been reported from Tokyo and its surrounding areas, as well as from Amami-Oshima, Japan, and Taiwan (Tokiwa et al., 2012, 2013) and is distinct from lineage ac5, which predominates on Okinawajima and Tokunoshima Islands (**Figure 1**) (Tokiwa et al., 2012, 2013; Sakashita et al., 2025). The fungal rRNA region (559 bp) was amplified and sequenced. BLAST analysis of the ITS2 region (171 bp) showed 100% sequence identity with *Aspergillus* species belonging to the *A. nomiae* clade including *A. nomiae* and *A. pseudonomiae* (Varga et al., 2011).

Based on these findings, the cause of death was diagnosed as neuroangiostrongyliosis and necrotizing pneumonia due to pulmonary angiostrongylosis with secondary aspergillosis.

## 4 Discussion

While the Ryukyu long-furred rat has previously been considered primarily susceptible to pulmonary angiostrongylosis, the present findings demonstrate that this endemic species can also develop severe neurological disease associated with meningeal infection. This suggests that insular rodent hosts may exhibit broader pathological responses to *A. cantonensis* than previously recognized, potentially influenced by host–parasite coevolution, population isolation, or exposure to distinct parasite lineages. Such lineage-specific host responses may have important implications for disease-mediated conservation risks in island-endemic mammals.

In permissive hosts of *A. cantonensis*, such as the Norway rat (*Rattus norvegicus*), larvae ingested with intermediate hosts migrate from the intestine to the brain and subsequently to the pulmonary arteries to mature, typically without causing neurological symptoms (Griffin et al., 2025; Spratt, 2015). In contrast, non-permissive hosts, including humans, develop neurological signs due to eosinophilic inflammation following larval death in the brain (Wang et al., 2015; Aghazadeh et al., 2016). To date, detection of *A. cantonensis* in the brain of the Ryukyu long-furred rat has been reported only once, from a single individual on Tokunoshima Island (Sakashita et al., 2025). In that case, larvae were detected in the brain, but neurological symptoms were not evident during life. In contrast, in the present case, nematode parasitism and marked lesions in the meninges were consistent with the neurological abnormalities observed prior to death. The absence of eosinophils in the present case may indicate that this individual exhibited a permissive-host-like response, or it may reflect the influence of prednisolone treatment. Consequently, the larvae did not die in the brain, and the present case showed both non-permissive-host–type (neurologic) and permissive-host–type (pulmonary arterial) disease patterns.

In humans, when *A. cantonensis* invades the central nervous system, analgesics are administered to relieve pain associated with meningitis, and corticosteroids are used to suppress inflammation (Ansdell et al., 2021). Anthelmintics such as albendazole are sometimes used, but larval death can induce systemic inflammatory reactions, and concurrent administration of corticosteroids is recommended (Chotmongkol et al., 2006). In the present case, prednisolone was administered but clinical improvement was not observed, likely reflecting an advanced stage of infection.

In the present case, extensive coagulative necrosis was observed throughout the lungs, with marked proliferation of filamentous fungi consistent with *Aspergillus* at the center of the necrotic foci. Fungal elements were also frequently detected within pulmonary arterial thrombi containing cross-sections of nematodes, suggesting that this fungal pneumonia developed secondary to the progression of the nematode infection. Adult *A. cantonensis* parasites inhabit the pulmonary arteries, where they induce vasculitis and thrombus formation. This likely resulted in impaired blood flow to the lung tissue and may have contributed to localized suppression of immune responses. Consequently, *Aspergillus* may have proliferated opportunistically and caused necrotizing pneumonia. Among the *A. nomiae* and closely related species, *A. nomiae* has been isolated from environmental sources and occasionally from clinical specimens (Zhou et al., 2020); however, its ecological distribution and pathogenic potential have not been fully elucidated. In addition, prednisolone administered to suppress inflammation associated with the nematode infection may have induced systemic immunosuppression, potentially exacerbating the fungal pneumonia. Taken together, these findings suggest that the cause of death in this case is presumed to be the combined effects of meningoencephalitis due to *A. cantonensis* and secondary fungal pneumonia.

Epidemiological information on *A. cantonensis* on Amami-Oshima Island remains limited. To date, infections have been reported only in the black rat (*Rattus rattus*) (Tokiwa et al., 2012) and the Ryukyu long-furred rat (Tokiwa et al., 2020), with no confirmed records from intermediate hosts (Noda et al., 1985). From a biogeographic perspective, the present findings further highlight the distinct distribution of *A. cantonensis* lineages within the Ryukyu Islands. To date, the ac1 haplotype has been documented on Amami-Oshima Island (Tokiwa et al., 2012, 2020), whereas the ac5 lineage predominates on Okinawajima and Tokunoshima Islands (**Figure 1**) (Tokiwa et al., 2012; Sakashita et al., 2025). The haplotype composition of *A. cantonensis* on other islands within the archipelago remains largely unknown. The identification of ac1 in the present case, together with previous records from Amami-Oshima Island, suggests that this lineage may be widely established and circulating on the island. Such lineage segregation among islands likely reflects a combination of historical colonization events, host movement, and island-specific ecological conditions. Further surveys of definitive and intermediate hosts across the Ryukyu Islands are needed to clarify the geographic boundaries of ac1 and ac5 lineages and to assess whether lineage-specific differences in host permissiveness or pathogenicity contribute to disease-mediated conservation risks in insular mammals.

Ryukyu long-furred rats are omnivorous and consume a wide range of prey items, including terrestrial gastropods such as *Bradybaena circulus*, *Coniglobus mercatorius*, *Meghimatium* sp., and *Satsuma eucosmia* (Stylommatophora), as well as *Cyclophorus turgidus angulatus* (Architaenioglossa). In addition, amphibians such as *Buergeria japonica* and *Zhangixalus viridis* (Anura), *Cynops ensicauda popei* (Caudata), and reptiles such as *Takydromus smaragdinus* (Squamata) have also been documented in its diet (Kudatka and Kudaka, 2017; Kobayashi et al., 2024). Although these dietary data are derived from the Okinawan population, a broadly similar diet may be expected in the Amami-Oshima population. Among these prey items, terrestrial gastropods may serve as intermediate hosts, whereas amphibians and reptiles could potentially act as paratenic hosts (Turck et al., 2022). Therefore, investigations of infection in these potential hosts, together with the management of invasive rodents, will be essential for understanding transmission dynamics and mitigating disease-mediated conservation risks on Amami-Oshima Island.

## Data Availability

The datasets presented in this study can be found in online repositories. The names of the repository/repositories and accession number(s) can be found below: https://www.ncbi.nlm.nih.gov/, LC909060 https://www.ncbi.nlm.nih.gov/, LC909059.
